# Three-Step Method for Proliferation and Differentiation of Human Embryonic Stem Cell (hESC)-Derived Male Germ Cells

**DOI:** 10.1371/journal.pone.0090454

**Published:** 2014-04-01

**Authors:** Jung Jin Lim, Myung Sun Shim, Jeoung Eun Lee, Dong Ryul Lee

**Affiliations:** 1 Fertility Center of CHA Gangnam Medical Center, College of Medicine, CHA University, Seoul, Korea; 2 Department of Biomedical Science, College of Life Science, CHA University, Seoul, Korea; Konkuk University, Korea, Republic of

## Abstract

The low efficiency of differentiation into male germ cell (GC)-like cells and haploid germ cells from human embryonic stem cells (hESCs) reflects the culture method employed in the two-dimensional (2D)-microenvironment. In this study, we applied a three-step media and calcium alginate-based 3D-culture system for enhancing the differentiation of hESCs into male germ stem cell (GSC)-like cells and haploid germ cells. In the first step, embryoid bodies (EBs) were derived from hESCs cultured in EB medium for 3 days and re-cultured for 4 additional days in EB medium with BMP4 and RA to specify GSC-like cells. In the second step, the resultant cells were cultured in GC-proliferation medium for 7 days. The GSC-like cells were then propagated after selection using GFR-α1 and were further cultured in GC-proliferation medium for 3 weeks. In the final step, a 3D-co-culture system using calcium alginate encapsulation and testicular somatic cells was applied to induce differentiation into haploid germ cells, and a culture containing approximately 3% male haploid germ cells was obtained after 2 weeks of culture. These results demonstrated that this culture system could be used to efficiently induce GSC-like cells in an EB population and to promote the differentiation of ESCs into haploid male germ cells.

## Introduction

Mouse and human embryonic stem cells (ESCs), which are derived from the inner cell mass of the blastocyst, have the capacity to self-renew and differentiate into all three germ layers [1]–[2]. ESCs can also spontaneously differentiate into primordial germ cell (PGC)-like cells and advanced germ cells *in vitro* [3]–[5]. The *in vitro* generation of sperm cells and oocytes from ESCs is beneficial for the basic and clinical study of reproduction.

Millions of mature sperm are produced from spermatogonia during spermatogenesis. These spermatogonia originate from PGCs in the genital ridge [6]. Genetic analysis using targeted mutations and co-culture has revealed that bone morphogenic protein (BMP) signaling is required for the generation of PGCs from early embryonic stage cells [7]–[10]. In addition, retinoic acid (RA), which regulates the transcriptional activity of various target genes, has been shown to induce the differentiation of PGCs into germ (spermatogonial) stem cells (GSCs, self-renewed spermatogonia) or differentiated spermatogonia [11]–[12]. Putative PGCs, derived from mouse ESCs through BMP and/or RA induction, have been found to produce sperm with morphological characteristics after transplantation into busulfan-treated adult testis or haploid male gametes with fertilizing ability *in vitro* [4], [8]. In humans, germ cell differentiation from ESCs via spontaneous or BMP-induced embryoid body (EB) formation has been reported [9], [13], and these studies revealed that human ESCs are capable of forming germ cells *in vitro*. However, the efficiency of this differentiation is low, and a specific protocol using BMPs and RA for differentiation into germ cells has not been well-established.

Three-dimensional (3D)-culture systems based on biomaterials are important tools for studying cell proliferation, differentiation and regeneration. Alginate is one of the biomaterials used in 3D-culture systems. Alginate is a natural polysaccharide obtained from brown seaweed, which forms a physical hydrogel in the presence of divalent cations, such as calcium [14]. Alginate is stable against mammalian enzymatic digestion but can be eliminated by *in vivo* mechanisms [15]. Owing to the biocompatibility, the mildness of gelation conditions and the low immunogenicity of purified alginate, this compound has been widely used in biomedical, biomaterial and therapeutic applications [16].

The purpose of this study was to refine the induction protocol for enriching GSC-like cells (induced spermatogonia) from human ESCs, to characterize the gene expression of these cells compared with testicular spermatogonia, and to develop an efficient 3D-co-culture system using alginate encapsulation and testicular somatic cells for differentiation into advanced male germ cells.

## Materials and Methods

### Culture of Human ESCs

This study was undertaken under approval of the Institutional Review Board for Human research of CHA Gangnam Medical Center, Seoul, Korea and the National IRB board regarding the research using human ESCs. Protocols for the use of animals in these experiments were approved by the Institutional Animal Care and Use Committee of CHA University (IACUC). Human ESCs (CHA-hES15: hES12010028, 40∼50 passages, Korea Stem Cell Registry, KNIH, Osong, South Korea; and H1: WiCell, 70∼80 passages, Madison, WI; both normal 46XY) were maintained according to a previously described method [17]. Briefly, undifferentiated human ESCs were maintained on mitomycin-C treated mouse embryonic fibroblast (MEF) cells in ES medium [DMEM/F12 (Invitrogen, Grand Island, NY) supplemented with 20% (v/v) knockout serum replacement (KSR, Invitrogen), penicillin (100 IU/mL, Welgene, Daegu, South Korea), streptomycin (100 µg/mL, Welgene), 0.1 mM non-essential amino acids (Invitrogen), 0.1 mM *β*-mercaptoethanol (Invitrogen) and 4 ng/mL basic fibroblast growth factor (bFGF, Invitrogen)]. The medium was changed daily. For the maintenance of undifferentiated human ESCs, the ESCs cultures were mechanically passaged weekly and were subsequently transferred onto freshly prepared MEF cells. The cell lines were cultured at 37°C in 5% CO_2_.

### The first step (Induction of GSC-like cells from ESCs)

ESCs were differentiated in EB medium (DMEM/F12 medium) containing 10% fetal bovine serum (FBS), 0.5-fold N2 and B27 (Invitrogen), 0.05% bovine serum albumin (BSA, Sigma), 2 mM GlutaMAX (Invitrogen), 0.5 mM ascorbic acid (Sigma) and 4.5×10^−5^ M 1-thioglycerol (Sigma) [18]. After trypsinization, the disassociated ESCs were seeded onto gelatin-treated plates for 40 min to remove the feeder cells. The unattached cells were subsequently cultured in EB medium for 72 hr [19]. After 72 hr, the specification into GSC-like cells from EBs was induced using either treatment with 100 ng/ml BMP4 and/or 0.1 µM RA. Those four groups (EB medium, EB medium+BMP4, EB medium+RA or EB medium +BMP4/RA group) were cultured for 2–8 days at 37°C in 5% CO_2_.

### The second step (Expansion of GSC-like cells)

After 4 days culture, GSC-like cells from EB medium+BMP4/RA group were cultured in EB culture medium or still EB medium+BMP4/RA or germ cell (GC)-proliferation medium [StemPro-34 SFM (Invitrogen)] supplemented with 10 µg/ml insulin-transferrin-selenium solution (ITS; Gibco), 6 mg/ml D-(+)-glucose, 30 µg/ml pyruvic acid, 1 µl/ml DL-lactic acid (Sigma), 5 mg/ml BSA (ICN Biomedicals, Costa Mesa, CA), 2 mM L-glutamine, 5×10^−5^ M 2-mercaptoethanol, MEM vitamin solution (Invitrogen), MEM non-essential amino acids solution (Invitrogen), 10^−4^ M ascorbic acid, 10 µg/ml d-biotin, 30 ng/ml β-estradiol, 60 ng/ml progesterone (Sigma), 20 ng/ml mouse epidermal growth factor (EGF; Becton Dickinson, Bedford, MA), 10 ng/ml human basic fibroblast growth factor (bFGF; Becton Dickinson), 10^3^ units/ml murine leukemia inhibitory factor (LIF; ESGRO, Invitrogen), 10 ng/ml Recombinant rat glial-derived neurotrophic factor (GDNF; R&D Systems, Minneapolis, MN) and 1% fetal calf serum (JRH Biosciences, Lenexa, KS) [20]. The cultures were maintained under the same conditions for an additional week. The medium was changed every 2 days. To collect and propagate GSC-like cells, the EBs were dissociated and sorted using a GFR-α1 antibody (Chemicon, Billerica, MA) through a magnetic activated cell sorting system (MACS; Dynal Flowcomp, Invitrogen). MACS-sorted GSC-like cells were additionally cultured in GC-proliferation medium for 3 weeks. Cultured cells were divided into two groups. One group was used for differentiation into advanced germ cells, and the other group was used for global gene expression analysis.

### The third step (*In vitro* differentiation of GSC-like cells into haploid germ cells using modified 3D-co-culture)

After culture, GSC-like cells from GC-proliferation medium group were harvested. Enzyme-dissociated cells were resuspended at 1×10^9^ cells/ml in DMEM/F12 medium containing 0.5% bovine calf serum (Hyclone) and 50 µg/ml of phytohemagglutinin (Sigma). After incubation for 10 min at 37°C, the cells were centrifuged, and the supernatant was removed. The aggregated cells were extruded into 1 ml sodium alginate solution (0.01 mg/ml in saline, custom-made RGD-coupled alginate with high glucouronic acid content, NovaMatrix FMC Biopolymer) in a Petri dish using a fire-polished Pasteur pipette [200 µm outside diameter (o.d.)]. The extruded strands of alginate-treated cells were drawn with a column of alginate solution into the tip of a 9″ Pasteur pipette (1 mm o.d.) and transferred into a calcium chloride solution (0.015 mg/ml in saline, Sigma) [21]–[22]. The alginate-encapsulated cell aggregates were co-cultured with and without testicular somatic cell (10^4^/ml) in differentiation medium compromising HEPES-buffered DMEM/F-12 medium supplemented with 10 µg/ml ITS solution, 10^−4^ mol/l vitamin C (Sigma), 10 µg/ml vitamin E (Sigma), 3.3×10^−7^ mol/l retinoic acid (Sigma), 3.3×10^−7^ mol/l retinol (Sigma), 1 mmol/l pyruvate (Sigma), 2.5×10^−5^ IU recombinant human FSH (Gonal-F; Merck-Serono, Modugno Bari, Italy), 10^−7^ mol/l testosterone (Sigma), 1× antibiotic-antimycotic solution (ABAM, containing penicillin, streptomycin and amphotericin B; Gibco/Invitrogen) and 10% bovine calf serum (Hyclone) in a 24-well dish and cultured for up to 6 weeks at 32°C in a humidified atmosphere of 5% CO_2_. The medium was replaced every other day. After 2 weeks of *in vitro* differentiation, the differentiated germ cells were analyzed through immunocytochemistry, RT-PCR, FISH and flow cytometric analysis. These 3-step methods were summarized in supporting information (Fig. S1).

### Testis tissue samples and cell preparation

This study was approved by the Institutional Review Board for the Human research of CHA Gangnam Medical Center, Seoul, South Korea. All donors for testicular tissues were understood the scope of the study and participated in this study by providing their written informed consent. There was no monetary compensation for their donations. Human fetal testis was obtained following elective termination of pregnancy (18 weeks gestation). Adult testicular tissues were obtained from obstructive azoospermic (OA) patients (with normal spermatogenesis) undergoing testicular sperm extraction (TESE)–intracytoplasmic sperm injection (ICSI) treatments. The testicular material remaining after clinical requirements was donated for the isolation of male GSCs. Human testis-derived GSCs were isolated and propagated using a previously described protocol [23]. For the co-culture of testicular somatic cells, the dissociated testicular cells obtained from TESE were cultured in GC-proliferation medium for 2 days. The medium was changed every other day, and the unattached germ cells and cell debris were discarded. After 2 passages, the cells were counted and cryo-preserved until further use.

### Reverse transcriptase-polymerase chain reaction (RT–PCR)

RT–PCR was performed to assess the expression of stage-specific marker genes (*OCT4 and NANOG* for ESCs; *VASA*, *Integrin α6* and *Integrin β1* for GSC-like cells or spermatogonial cells; c-Kit for spermatogonia and spermatocytes; testis-specific histone protein 2B (TH2B) for spermatocytes; transition protein (TP-1) for spermatids) in 3D-co cultured cells induced from ESCs (CHA-hES15 and H1 cell lines). Total RNA was extracted from the cultured cells using the TRIzol method (Gibco). Reverse transcription was performed using 1 µg of total RNA, 5 mmol/l MgCl_2_, and 1 IU of DNase I at 37°C for 30 min followed by the addition of 1 mmol/l dNTP, 2.5 µmol/l oligo-dT, and 2.5 IU reverse transcriptase (Superscript, Invitrogen) and incubation at 42°C for 1 hr [24]. After the reaction was complete, the cDNA were either directly used for PCR or stored at −20°C. The following targets were amplified from cDNA using the primers indicated in parentheses: *OCT4* (F: 5′-GAA AGG CTT CCC CCT CAG GGA A-3′ R: 5′-AAG AAC ATG TGT AAG CTG CGG-3′; 460 bp; GenBank accession number NM 002701), *NANOG* (F: 5′-ATG CAG GCA ACT CAC TTT AT-3′ R: 5′-TTC AGG ATG TTG GAG AGT TC-3′; 548 bp; GenBank accession number NM024865), *VASA* (F: 5′-AAG AGA GGC GGC TAT CGA GAT GGA-3′ R: 5′-CGT TCA CTT CCA CTG CCA CTT CTG-3′; 238 bp; GenBank accession number NM024415), *Integrin α6* (F: 5′-GGG AGC CTC TTC GGC TTC TC-3′ R: 5′-CAC ATG TCA CGA CCT TGC CC-3′; 286 bp; GenBank accession number NM000210), *Integrin β1* (F: 5′-CTG CAA GAA CGG GGT GAA TG-3′ R: 5′-CAC AAT GTC TAC CAA CAC GCC C-3′; 301 bp; GenBank accession number BC020057) and 18S ribosomal RNA (F: 5′-TAC CTA CCT GGT TGA TCC TG-3′ R: 5′-GGG TTG GTT TTG ATC TGA TA-3′; 255 bp; GenBank accession number K03432). The amplification was performed in a 20-µl reaction mixture containing 10 mmol/l Tris–HCl (pH 8.3), 2 mmol/l MgCl2, 50 mmol/l KCl, 0.25 mmol/l dNTP, 3–5 pmol of each primer, and 1.25 IU Taq polymerase (Gibco). The PCR was initiated with denaturation at 94°C for 5 min followed by 35–40 cycles of 30 sec at 94°C, 30 sec at 55–60°C, and 30 sec at 72°C with a final extension step for 10 min at 72°C. The PCR products were confirmed on a 1.5% agarose gel and visualized under the UV light after ethidium bromide staining. The negative controls included mock transcription without mRNA.

### Immunocytochemical analysis

To investigate the localization and expression of ESC and GSC markers in cultured colonies or MACS-sorted cells, we performed immunocytochemistry using antibodies [OCT4 (Santa Cruz Biotechnology, Santa Cruz, CA), SSEA-4 (Chemicon), TRA-1-60 (Chemicon) and SSEA-3 (Chemicon) were used for ESC; integrin α6 (Santa Cruz Biotechnology) and integrin β1 (Santa Cruz Biotechnology) were used for ESC or GSC; VASA (R&D Systems) and GFR-α1 (Chemicon) were used for GSC or GSC-like cells; intra-acrosomal protein (IAP, Abcam) was used for spermatid [25]. The samples were washed three times in DPBS containing 5% FBS and subsequently fixed in paraformaldehyde (4% v/v in DPBS) for 24 hr. For permeabilization, the cells were incubated in 0.1% Triton X-100 in DPBS for 10 min. After washing three times with DPBS, non-specific antibody binding was suppressed through incubation in blocking solution (4% normal goat serum in DPBS) for 30 min at room temperature. After additional washing three times with PBS, the fixed samples were subjected to immunocytochemical staining through incubation with a primary antibody diluted to 1∶200–1∶500 with DPBS containing 1% BSA for 60 min at room temperature or overnight at 4°C. The immunoreactive protein was subsequently detected using CY3 or FITC-conjugated secondary antibodies diluted to 1∶500 with DPBS for 60 min at room temperature. The samples were counterstained using 1 µg/ml 4′,6-diamidino 2-phenyindiol (DAPI; Sigma). Following multiple washes, the cells were mounted in Vectashield mounting medium (Vector Laboratories, Burlingame, CA). The stained cells were viewed on an inverted confocal laser scanning microscope (LSM 510; Carl Zeiss, Oberkochen, Germany) with fluorescence at 400× magnification. The micrographs were stored in LSM (Zeiss LSM Image Browser version 2.30.011; Carl Zeiss Jena GmbH, Jena, Germany). Negative control was performed by using the isotype IgG instead of primary antibody or protein Block (DAKO Corporation, Carpinteria, CA) without primary antibody. Percentage of VASA-positive cells in EBs derived from CHA-hES15 and H1 hESC lines were evaluated with a counting of 200 cells per each slide.

### Fluorescence in situ hybridization (FISH)

FISH was performed to confirm the haploidy of presumptive spermatids derived from 3D co-cultured GSC-like cells. After 2 weeks of *in vitro* differentiation, the differentiated germ cells were dispersed and placed in hypotonic solution [6 mg/ml bovine serum albumin and 0.5% sodium citrate (Sigma)] for 10 min, and fixed in Carnoy's solution (methanol:acetic acid = 3∶1) for 10 min. Next, fixed cells were spread onto cleaned glass slides, dehydrated, and examined with directly labeled DNA probes (Vysis Inc., Framingham, MA, USA), Chromosome 9 (spectrum red) and Chromosome X (spectrum green). The probe mixture was applied to the slide and covered with a cover glass. Probe and target cells were co-denatured by heating the slide to 73°C for 10 min. Hybridization was carried out in a moist chamber at 37°C for overnight. Following hybridization, the slides were washed using 0.4× SSC/0.3% NP40 solution at 73°C for 3 min. The slides were air-dried, counterstained using DAPI and mounted. Following FISH, the nuclei and fluorescence signals were viewed using a fluorescence microscope.

### Cell proliferation assay

Proliferations of PGC-like cell were assessed using PrestoBlue assay kit (Invitrogen) according to the manufacturer's protocol. After 1 or 4 weeks culture, the cells were dissociated and seeded at 1×10^4^ cells/well in a 96-well plate for 24 hr. The cells were washed and incubated with PB reagent for 2 hr. The proliferation activities were detected using fluorescence spectroscopy. The fluorescence was read (excitation 560 nm; emission 590 nm). The cell proliferation was expressed as a relative fold change.

### Flow cytometric analysis

Flow cytometric analyses were performed using a standard protocol. Induced GSC-like cells, dissociated in trypsin-EDTA, and aliquots of 10^6^ cells were resuspended in 0.1 ml of PBS containing 2% fetal bovine serum (PBS/FBS) and subsequently incubated with primary antibodies. To quantify GSC-like cells, the cells were incubated with 10 mg/ml of GFR-α1 antibody (Chemicon). The primary antibodies were detected using 5 mg/ml of a FITC-conjugated secondary antibody. The control cells were not treated with primary antibodies. The cells were maintained in the dark on ice until analysis on a Becton Dickinson FACS IV Calibur (Becton Dickinson, San Jose, CA). At least 5000 to 10000 events were acquired for each sample.

To analyze the DNA content after germ cell differentiation, the encapsulated cells were dissociated in trypsin-EDTA and resuspended in DPBS to release single cells. After filtering through 80-µm nylon mesh, the cells were fixed in cold 70% ethanol and maintained at 4°C until further analysis. For the DNA content assay, 1×10^6^ cells were washed twice with DPBS and incubated in 500 µL of 0.2% pepsin for 10 min at 37°C. After centrifugation, the cells were stained in a solution containing 25 µg/ml propidium iodide (PI) (Sigma), 40 µg/ml RNase (Gibco/Invitrogen), and 0.3% Tween-20 in PBS at room temperature for 20 min. The stained cells were analyzed using a FACS IV system.

### Transplantation of ESC-derived GSC-like cells into seminiferous tubule of W/W^V^mice

Cultured ESC-derived GSC-like cells were transplanted into ten dominant-white spotting (W) locus mutant recipient mice (W/W^v^; The Jackson Laboratory, Bar Harbor, ME) in the first (3 mice), second (3 mice) and third (4 mice) experiments. To transfer the cells into the seminiferous tubules of a recipient testis, we used a transfer technique via the efferent duct [26]. GSC-like cells derived from genetically modified human ESCs (CHA-hES3-GFP, hES12010001: Korea Stem Cell Registry, KNIH, Osong, South Korea, normal 46XY, [27]) were dissociated and suspended in DPBS medium containing 0.4% trypan blue (Sigma). The W/W^v^ recipient mouse was anesthetized, and the testis was exteriorized through a midline abdominal incision and immobilized. The injection pipette was constructed from a three-inch length of borosilicate glass with an internal diameter of 0.75 mm and an external diameter of 1 mm (World Precision Instruments # TW100-3). The glass was drawn on a Kopf pipette puller (Model 750) creating two potential injection pipettes [40–60 µm outside diameter (o.d.)]. The glass pipette was inserted into the efferent duct under a dissecting microscope, and the pressure in the injection tubing was raised until the cell suspension flowed into the seminiferous tubule. The flow was monitored based on the color change. A cell suspension of approximately 3–5 µl (less than 1×10^5^ cells) was injected into a W/W^v^ recipient testis, filling ∼50% of the tubules in each testis. One to three weeks after transplantation, the recipient testes were collected, detunicated and analyzed for GFP expression using a fluorescent microscope.

### Microarray and data analysis

Each total RNA sample (200 ng) obtained from the undifferentiated hESC, hESC-derived GSC-like cells and testis-derived spermatogonia were labeled and amplified using the Low Input Quick Amp Labeling kit (Agilent Technologies, Palo Alto, CA). The Cy3-labeled RNAs were resuspended in 50 µl of hybridization solution (Agilent Technologies). After the labeled RNAs were placed in an Agilent SurePrint G3 Human Gene Expression 8×60K Microarray (Agilent Technologies) and covered with a Gasket 8-plex slide (Agilent Technologies), the slides were hybridized for 17 hr at 65°C. The hybridized slides were washed in 2× SSC, 0.1% SDS for 2 min, 1× SSC for 3 min, and 0.2× SSC for 2 min at room temperature. The slides were centrifuged at 3000 rpm for 20 sec to dry. The arrays were analyzed on an Agilent scanner using the associated software. The gene expression levels were calculated using Feature Extraction v10.7.3.1 (Agilent Technologies). The relative signal intensities for each gene were generated using the Robust Multi-Array Average algorithm. The data were processed based on the median polish normalization method using GeneSpring GX 7.3.1 (Agilent Technologies). This normalization method mediates the distribution of intensities for each array in a set of similar arrays. The normalized and log-transformed intensity values were subsequently analyzed using GeneSpring GX 7.3.1 (Agilent Technologies). The fold-change filters included a requirement that the genes are present in at least 200% of the controls for up-regulated genes and lower than 50% of the controls for down-regulated genes. The hierarchical clustering data comprised clustered groups with similar characteristics across experiments using GeneSpring GX 7.3.1 (Agilent Technologies). The clustering algorithm represented the Euclidean distance and average linkage. Microarray data have been deposited in Gene Expression Omnibus (GEO) database, www.ncbi.nlm.nih.gov/geo/ (accession no. GSE52302).

### Validation of microarray results (Quantitative RT-PCR)

Quantitative RT-PCR was performed for the undifferentiated hESCs, GSC-like cells induced from hESCs and testicular spermatogonia using SYBR® green I reagents and the following probe sets: *TENC1* (F: 5′-CCT CTT TGC AGA GCT TGA CC-3′ R: 5′-CTG GCC CAG TAG AAC TTT GG-3′, 76 bp), *PTPRM* (F: 5′-GTG GCC CTG GAA TAC TTG AA-3′ R: 5′-GAA CGT CTT GGC TTT GTG GT-3′; 84 bp), *ENC1* (F: 5′-CTC GAA CTG CTT TCG TCC AT-3′ R: 5′-CCC AAC TAC AGC CCA CTC AT-3′; 198 bp), *NR0B1* (F: 5′-CTG TTC TTC AGG CCC ATC AT-3′ R: 5′-CTT CCC TCAT GG TGA ACT GC-3′; 124 bp), *EPB41L3* (F: 5′-AAA GAG CAG CAC CCT GAC AT-3′ R: 5′-CTA ACG GTT TGC ATG ACT GC-3′; 134 bp), *DUSP6* (F: 5′-TTG CTT GTG TTG TCG CAA A-3′ R: 5′-TGC ATT TGA GGT GAC ACT CC-3′; 150 bp), *NID2* (F: 5′-CCT ACT GCC CAA CAG GAA GA-3′ R: 5′-TGC CTT TGC AGT CAC TGT TC-3′; 100 bp), *STARD13* (F: 5′-CTG TAT GCC AGC ACA GGA GA-3′ R: 5′-CTC TTG GAG CCC ATT GAC AT-3′; 96 bp).

### Statistical analysis

All experiments were replicated at least 3 times, and the data are presented as the means ± SEMs. The statistical significance of the differences among treated groups was evaluated using one-way analysis of variance (ANOVA) with a log-linear model in the Statistical Analysis System (SAS, Cary, NC, USA). Values of P<0.05 were considered to be statistically significant.

## Results

### Characterization of hESCs and spermatogonia from human testis

Prior to the experiments, the expression of the pluripotent stem cell markers OCT4, NANOG, SSEA-3, SSEA-4, and TRA1-60 was reconfirmed in both of the undifferentiated human ESC lines: CHA-hES15 cells (Fig. S2) and H1 cells (data not shown). The expression of the GC-specific markers VASA and GFR-α1 was not observed in undifferentiated human ESC lines (Fig. S2). However, these markers were detected in the primordial germ cell and spermatogonia from the testes, respectively (Fig. S3A). In the fetal gonad, immunohistochemical staining revealed that the VASA protein was present in the primordial germ cell only. But, in the adult testis, VASA protein was present in the spermatogonia, spermatocytes and some mature spermatozoa (data not shown). The GFR-α1 protein was detected only in the spermatogonia located on the basement of a tubule (Fig. S3A), suggesting that this marker could be a more specific marker of spermatogonia. In addition, integrin α6 and β1 were expressed in both isolated testicular spermatogonia and human ESCs (Fig. S3B,C).

### The first step: Induction of GSC-like cells from hESC-derived EBs

**Figure 1:**
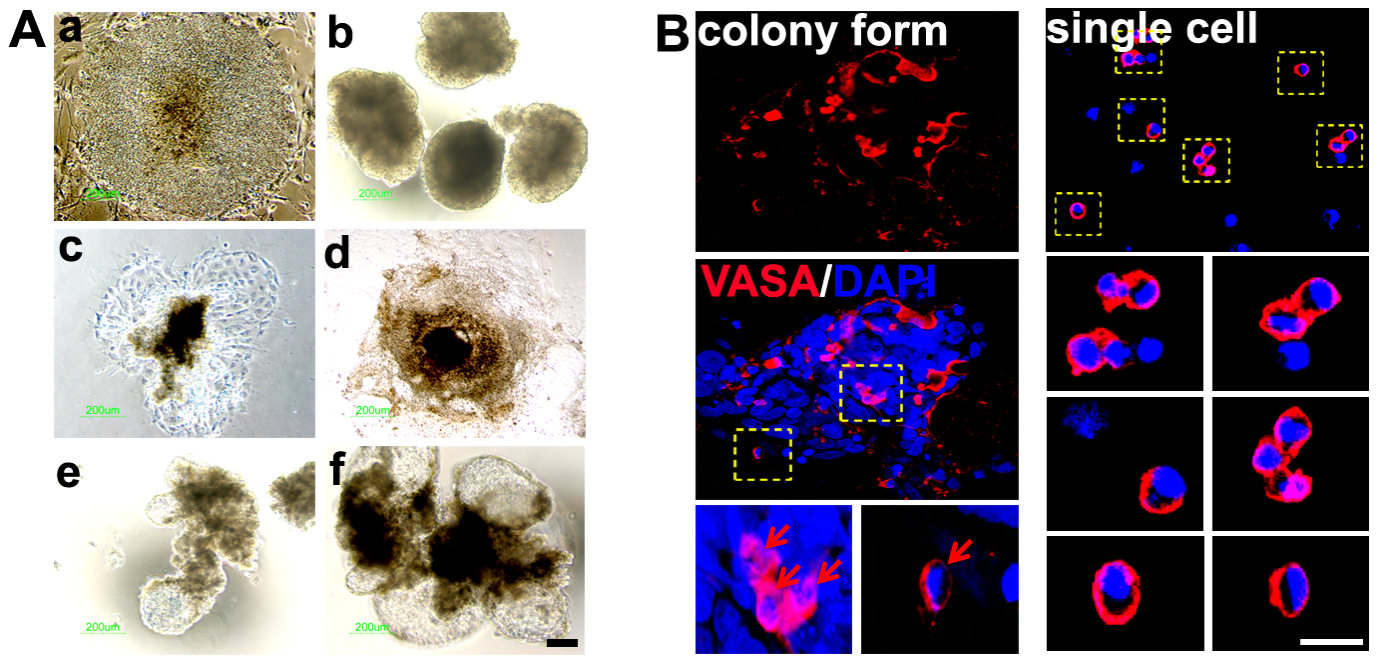
Differentiation of embryoid bodies (EBs) into male germ stem cell (GSC)-like cells. (A) Effects of treatment (EB medium; Non-treated, EB medium+BMP4, EB medium+RA or EB medium +BMP4/RA group) of EBs on the morphological features and induction of GSC-like cells after culture for 4 days. Scale bars: 50 µm. a; undifferentiated CHA hES 15, b: embryoid body from CHA hES 15, c; EB medium, d; EB medium+BMP4, e; EB medium+RA, f; EB medium +BMP4/RA group (B) Immunocytochemical staining using anti-VASA antibody on 4-day-old EBs (the left panel) and dissociated cells (the right panel). The yellow box indicates the magnified area. The red arrows indicate the signals for VASA fluorescence. **Note:** Scale bars are 20 µm.

**Figure 2:**
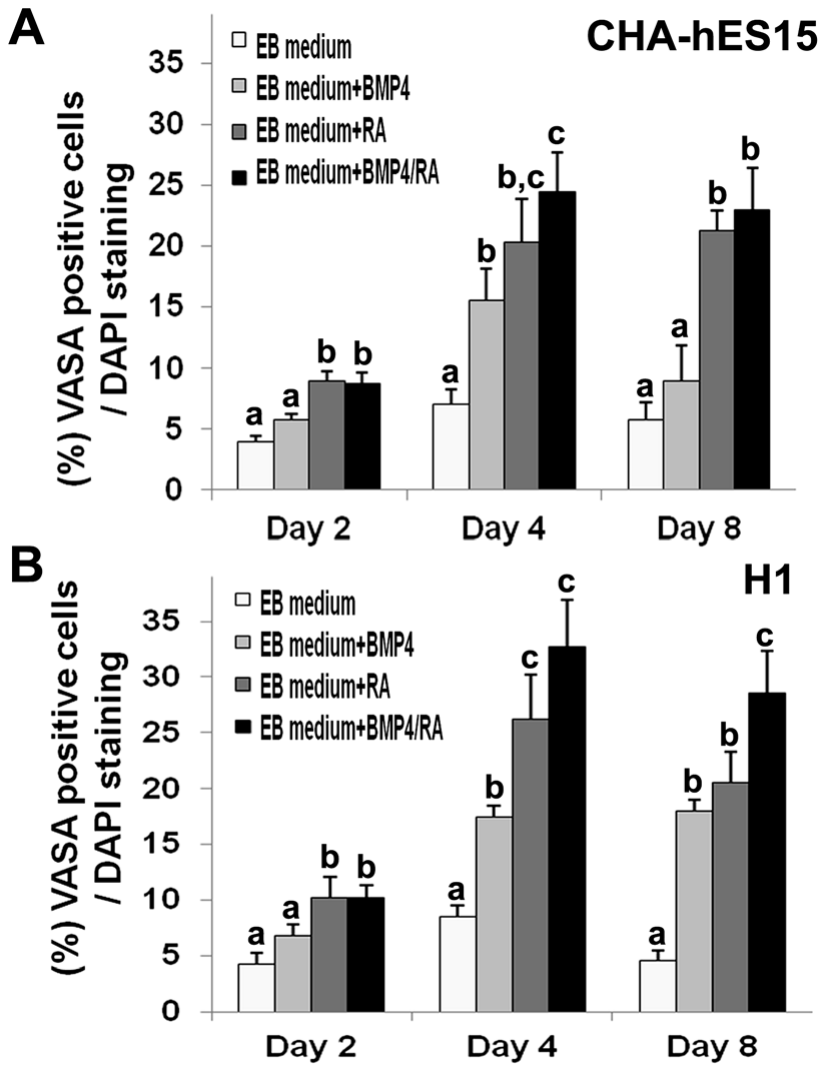
Graphic representations of stained VASA-positive cells. Percentage of VASA-positive cells in EBs derived from CHA-hES15 and H1 hESC lines treated with EB medium (non-treated), RA, BMP4 or RA+BMP4 for 2–8 days. RA: retinoic acid; BMP4: bone morphogenetic proteins 4. **Note:** ^a^ *vs* ^b^ *vs* ^c^, *p*<0.05.

When ESCs were cultured for 3 days in EB medium, three-dimensional structures called EBs were formed. For specification into the germ lineage, BMP4 and/or RA were added into the EB medium for 4 days. The EBs were attached and spread onto a culture dish in non-treated or BMP4-only treated groups. However, in the RA only- or BMP4/RA-treated groups, the EBs were clustered together at the periphery of the cells and were swollen (Fig. 1A). For effective induction of EBs into GSC-like cells, the specification rate into the GC lineage was compared based on the expression of VASA for 2–8 days after treatment of the cells with BMP4 and/or RA. The VASA protein was expressed on the membrane and in the cytoplasm of developing PGCs at the genital ridge [28]. Although there was no VASA expression in undifferentiated hESCs (Fig. S2), the VASA-positive cells were most frequently localized on the surface of EBs (Fig. 1B). Figure 2 shows the percentages of VASA-positive putative GSC-like cells. The results also indicated that the specification in CHA-hES15 and H1 cells could potentially be enhanced through treatment with BMP4 or RA, which are important for differentiation into GCs *in vitro*. On day 2, VASA was detected in EBs cultured with and without BMP4/RA. The percentage of VASA-positive cells in EBs cultured with RA (9.0%±0.7, 10.3%±1.8 in CHA-hES15 and H1) and BMP4/RA (8.8%±0.9, 10.3%±1.1) was significantly greater than that in EBs cultured without factors (4.0%±0.4, 4.3%±1.1) and that in the BMP4 (5.8%±0.5, 6.8%±1.1)-treated group (*p*<0.05). After 4 days, a significant increase in percentage of VASA positive cells was detected in EBs cultured with BMP4 (15.5%±2.6 and 17.5%±2.9 in CHA-hES15 and H1), RA (20.4%±3.6 and 26.3%±4.0) or BMP4/RA (24.5%±3.2 and 32.8%±4.2) compared with the EB medium group (7.0%±1.3 and 8.5%±1.7)(*p*<0.05). In particular, on day 4, the EBs treated with BMP4/RA showed the highest expression of VASA. The VASA expression on day 8 (EB medium group: 5.8%±1.4 and 4.5%±1.6 in CHA-hES15 and H1, BMP-treated: 9.0%±2.9 and 18.0%±3.4, RA-treated: 21.3%±1.7 and 20.5%±2.8, BMP4/RA-treated: 23.0%±3.5 and 28.5%±3.8) was not increased compared with that of the 4-day culture groups. Indeed, the VASA expression was reduced in certain groups, and there were slightly more dead cells in the EBs (data not shown). The number of putative GSC-like cells was higher in BMP4/RA-treated EBs than in the other groups. Thus, we decided to use BMP4/RA in further experiments.

### The second step: Proliferation and isolation of PGC-like cells from hESC-derived EBs

**Figure 3:**
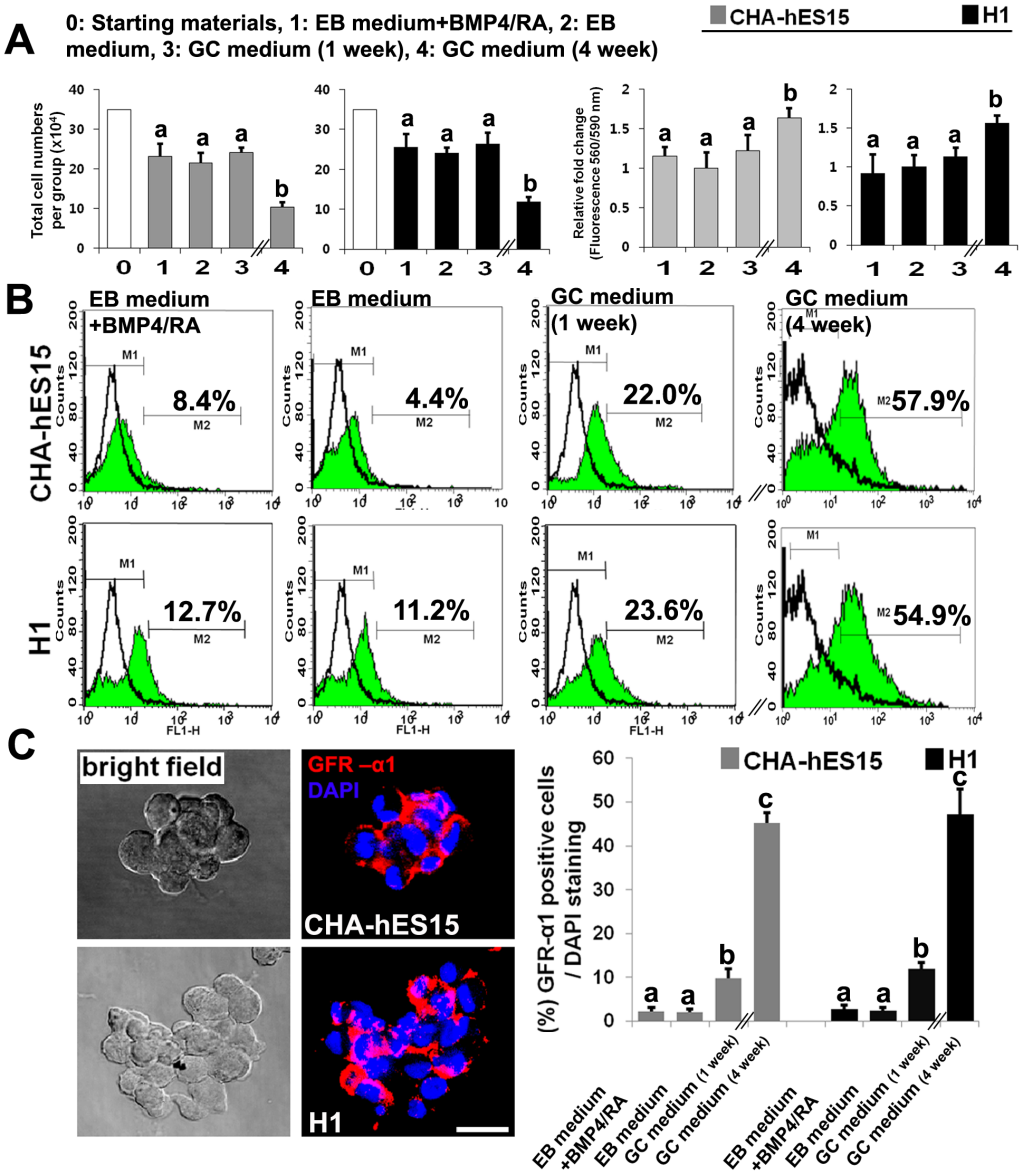
Proliferation of GSC-like cells through culture with GDNF, bFGF and LIF. (A) Total numbers and proliferation activity of embryoid body (EB) cells (from CHA-hES15 and H1 cell lines) in cultured groups with EB medium+BMP4/RA, EB medium only or GC proliferation medium for 1 and 4 weeks. (B) Flow cytometric analysis of GFR-α1 expression in ESC-derived GSC-like cells (from CHA-hES15 and H1 cell lines) cultured in EB medium+BMP4/RA, EB medium only or GC proliferation medium for 1 and 4 weeks. (C) Immunocytochemical staining of GFR-α1 expression in ESC-derived GSC-like cells (from CHA-hES15 and H1 cell lines) cultured in EB medium+BMP4/RA, EB medium only or GC proliferation medium for 1 and 4 weeks. **Note:** ^a^ *vs* ^b^ *vs* ^c^, *p*<0.05, Scale bars are 50 µm.

GC-proliferation medium was used to propagate GSC-like cells for 1 to 3 weeks. The population of GSC-like cells (induced spermatogonia) was analyzed based on the expression of GFR-α1, which is a more reliable marker of spermatogonia detection than VASA. Before the flow cytometric analysis, we have counted total cell number of each group (EB medium group+BMP4/RA, EB medium group and GC-medium). As shown in the Fig. 3A, total cell numbers after 1 week of culture were decreased compared to that of starting material, but not different among three groups. In cell proliferation assay to evaluate the proliferation activity of each groups (EB medium group, EB medium group+BMP4/RA and GC-medium), there were no significant differences in all 1 week-cultured groups. However, MACS and long-term cultured cell in GC-medium (GC-medium for 4 weeks) have greater proliferating activity than other groups and total number of sorted cell was increased more than 4 times (Fig. 3A). The flow cytometric analysis revealed that after 1 week of culture, the population of GFR-α1-positive cells in the GC-proliferation medium group was much higher than that in the EB medium group or the BMP4/RA group (CHA-hESC15: 22.0%±2.6 *vs.* 8.4%±0.6 and 4.4%±1.2, H1: 23.6%±3.3 *vs.* 12.7%±3.2 and 11.2%±1.5, *p*<0.05)(Fig. 3B). The group cultured in the GC-proliferation medium was sorted according to GFR-α1 expression and further cultured under the same conditions for 3 weeks. After 4 weeks of culture (2 more passages) in GC-proliferation medium, the percentage of GFR-α1-positive cells reached 57.9%±3.2 and 54.9%±5.3 in CHA-hES15 cells and H1 cells, respectively (Fig. 3B). In addition, immunocytochemical analysis re-confirmed that percentage of GFR-α1-positive cells in the GC-proliferation medium group was much higher than that in the EB medium group or the BMP4/RA group after 1 week of culture (CHA-hESC15: 9.8%±2.3 *vs.* 2.0%±0.7 and 2.3%±1.0, H1: 12.0%±1.5 *vs.* 2.5%±0.6 and 2.8%±0.9, *p*<0.05)(Fig. 3C). Moreover, after 4 week of culture, the percentage of GFR-α1-positive cells reached 45.3%±2.4 and 47.3%±5.7 in CHA-hES15 cells and H1 cells, respectively (Fig. 3C).

### The third step: *In vitro* differentiation of PGC-like cells using a 3D-coculture system

**Figure 4:**
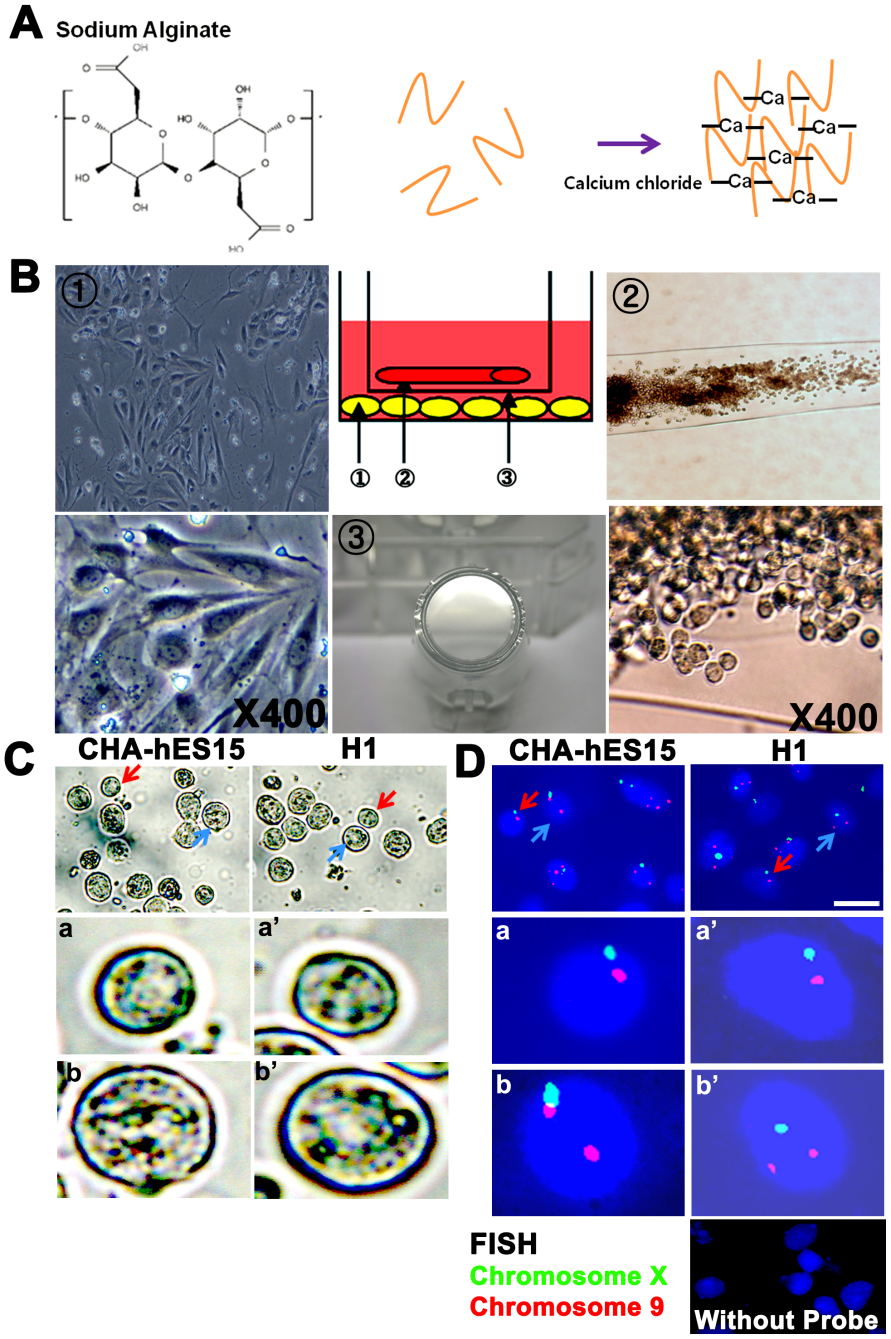
*In vitro* culture of GSC-like cells using a 3D-co-culture system and the potential for differentiation into germ cell lineagesI. (A) Schematic of calcium-crosslinked alginate; Soluble alginate in saline was mixed with CaCl_2_ solution. The diffusion of calcium ions into the surrounding solution induces the crosslinking of the soluble alginate and the formation of hydrogels. (B) 3D-co-culture system; The encapsulated GSC-like cells were passed through a strainer (insert), cultured in 6-well culture dishes and differentiated into testicular cells; (1) Human testicular cells (2) Encapsulated GSC-like cells (3) Strainer (insert) (C) Putative differentiated germ cell with a diameter of 15–25 µm. Red arrows, a and a′ indicated putative differentiated germ cell. And blue arrows, b and b′ indicated non-meiotic cell in 3D-co-culture group (from CHA-hES15 and H1 cell lines). (D) FISH analysis of *in vitro* differentiated GSC-like cells from hESCs; Red arrows, a and a′ indicated normal nucleus with meiotic cells (haploid; n) of chromosomes X (spectrum green) and 9 (spectrum red). And blue arrows, b and b′ indicated non-meiotic cells (diploid; 2n) of chromosomes X (spectrum green) and 9 (spectrum red) in 3D-co-culture group (from CHA-hES15 and H1 cell lines). Scale bars are 20 µm.

**Figure 5:**
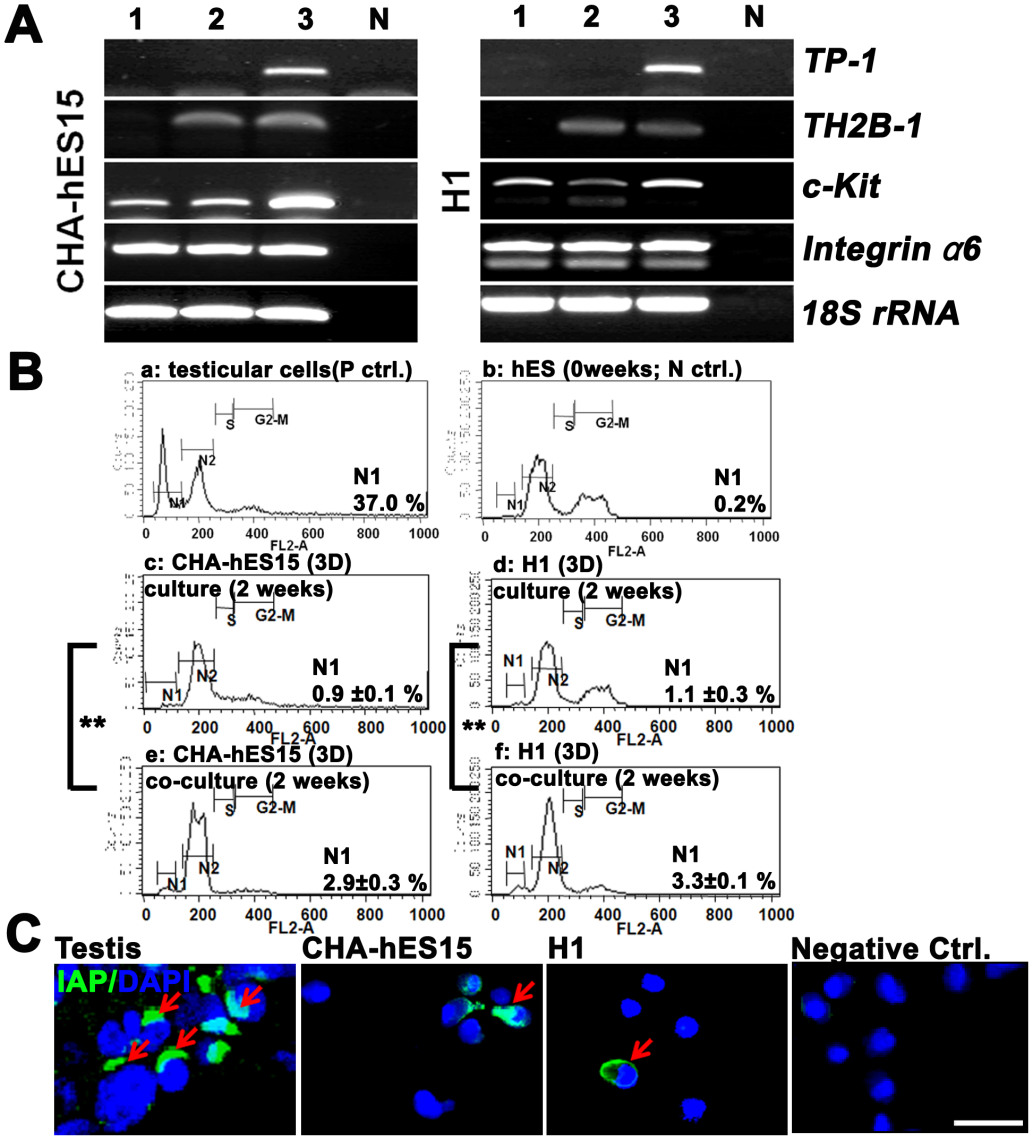
*In vitro* culture of GSC-like cells using a 3D-co-culture system and the potential for differentiation into germ cell lineages. (A) Expression of stage-specific genes (integrin α6, c-Kit, TH2B and TP-1) during *in vitro* differentiation of human ESC-derived GSC-like cells using RT-PCR. Integrin α6 (undifferentiated spermatogonia), c-Kit and TH2B (differentiating spermatogonia and spermatocytes), TP-1 (meiotic spermatocytes and spermatids) were used as stage-specific germ cell marker genes. 18S ribosomal RNA was used as a control gene. Line 1–3: ESC-derived GSC-like cells (0, 1, and 2-week culture in the 3D-co-culture system), N: no reverse transcriptase. (B) DNA content of *in vitro* differentiated ESC-derived GSC-like cells. a; DNA content of cells from obstructive azoospermia (OA, normal spermatogenesis sample) b; Undifferentiated human embryonic stem cells (CHA-hES15). N1 represents haploid germ cells and N2 represents diploid cells. (C) Localization of intra acrosomal protein (IAP) in human testes (upper panel) and *in vitro* differentiated GSC-like cells from hESCs (middle and lower figure). IAP was stained in spermatid acrosomes. The yellow arrows indicate fluorescence signals. **Note:** **; significantly different (*p*<0.05). Scale bars: 50 µm.

The structure of alginate-encapsulation containing GSC-like cells was not changed during *in vitro* differentiation (Fig. 4A and 4B). However, the populations of GSC-like cells in the inner wells and testicular cells in the outer wells increased. After 2 weeks of 3D-culture with or without co-culture with testicular cells, the encapsulated GSC-like cells were dissociated, and the morphology, FISH, gene expression and ploidy of these cells were analyzed. In the group co-cultured with testicular cells, although the numbers was very low, several types of GC-like cells ranging in size from 15 to 25 µm were appeared after *in vitro* culture. Their morphology was generally spherical with apparently intact plasma membranes and clear nuclear margins (Fig. 4C) and was looked similar to post-meiotic GC [22]. In FISH analysis, most of cultured cells showed non-meiotic (2n) cells and sperm-like cells were not identified. However, there were a few haploid (n) or tetraploid (4n) cells obtained after culturing (Fig. 4D and Fig. S7). In RT-PCR, TP-1 (a marker for spermatocyte and spermatid) expression and IAP (acrosome granule; a marker for spermatocyte and spermatid) expression were detected in differentiated germ cells derived from both types of ESCs (Fig. 5A and 5C). And integrin α6 (undifferentiated spermatogonia), c-Kit and TH2B (differentiating spermatogonia and spermatocytes) still remained in the 3D-co-culture group. The quantification analysis showed that the ratios of haploid germ cells were higher in the 3D-co-culture group than in the 3D-culture group (2.9±0.3% and 3.3±0.1% *vs.* 0.9±0.1% and 1.1±0.3% in the CHA-hES15 and H1, respectively, *p*<0.05) (Fig. 5B).

### *In vivo* differentiation (propagation) of PGC-like cells in the testes

To determine whether the ESC-derived GSC-like cells maintained their characteristics *in vivo*, the GSC-like cells were transplanted into recipient testes preceded by MACS sorting without *in vitro* differentiation. The transplantation of GSC-like cells resulted in the expression of GFP throughout the seminiferous tubule sections (Fig. S6). In 1–3 weeks after transplantation, the GSC-like cells derived from ESCs had a detectable GFP-positive population in some seminiferous tubules. At 3 weeks, an increase in GFP-positive cells was detected compared with at 1-week.

### Analysis of gene expression

We performed microarray analysis to identify the similarity of gene expression between ESC-derived GSC-like cells and testis-derived spermatogonia. Two sets of separately purified undifferentiated hESCs (CHA-hES4 and H1), cultured GSC-like cells (derived from CHA-hES4 and H1) and spermatogonia (derived from the testes of two individual OA patients) were used for mRNA preparation and microarray hybridization. Of the 42,404 genes on the microarray, 9,534 transcripts were expressed at a level above the threshold defining differential expression (Fig. S4). The clustering tree showed that the differences (>2-fold) in the expression patterns between the ESC-derived GSC-like cells and the testis-derived spermatogonia were less pronounced than those between the ESC-derived GSC-like cells and the undifferentiated hESCs (*p*<0.05) (Fig. S5).

**Figure 6:**
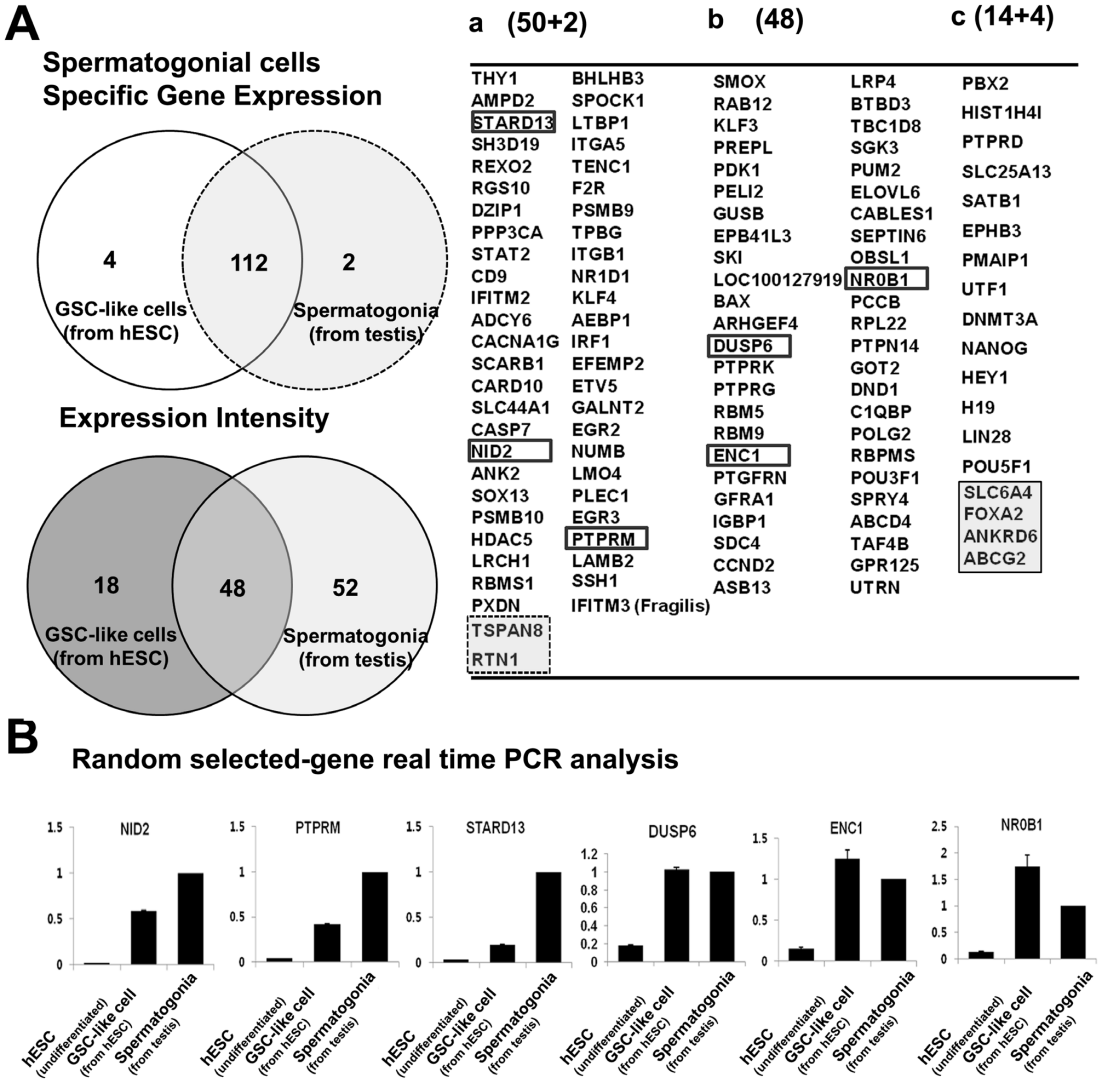
Comparative analyses between ESC-derived GSC-like cells and testis-derived spermatogonia using known spematogonial marker genes. (A) The expression profiles of the 118 genes in the 2 types of cells are presented as a Venn diagram and table. a; A total of 52 genes were more strongly expressed in testis-derived SSCs than in ESC-derived GSC-like cells. b; A total of 48 genes were similarly expressed in the 2 groups. c; A total of 18 genes were more strongly expressed in ESC-derived GSC-like cells than in testis-derived spermatogonia. (B) The validation of randomly selected spematogonial marker genes using real-time RT-PCR. **Note:** *In vitro* cultured ESC-derived GSC-like cells (at 2^nd^ passage); *In vitro* cultured testis-derived spermatogonia (at 2^nd^ passage).

To evaluate the germ cell lineage further, 140 marker genes were selected based on previously published data [29]. A total of 22 genes were then excluded because of similar expression in the undifferentiated hESCs (Fig. 6A). Of the 118 remaining genes, 112 genes were expressed by both the ESC-derived PGC-like cells and the testis-derived spermatogonia. The other six genes were expressed by only the ESC-derived PGC-like cells or the testis-derived SSCs. Forty-eight of the remaining 118 genes, including TAF4B, GPR-125, GFRA1, POU3F1, DND1, and SEPTIN6, showed similar expression levels in both ESC-derived GSC-like cells and the testis-derived spermatogonia, and 52 of the 118 genes, including INTG5, INTGB1, EGR2, THY-1, CD9, SOX 13, and IFITM3, were up-regulated in testis-derived spermatogonia compared with ESC-derived GSC-like cells. Only 18 of the 118 genes, including NANOG, POU5F1, LIN28, UTF1 and SATB1, were up-regulated in ESC-derived GSC-like cells compared with testis-derived spermatogonia (Figure 6A). We used quantitative RT-PCR to validate the differential expression of selected spermatogonia-specific genes in ESC-derived GSC-like cells compared with testis-derived spermatogonia. The RT-PCR analysis showed that the mRNA levels of selected genes, including STARD13, NID2, PTPRM, DUSP6, NCH1 and NR0B1, in the two groups were similar to the levels detected in the microarray results (Fig. 6B).

## Discussion

The pluripotency of ESCs provides an excellent model for studying differentiation and dedifferentiation in various lineages, including the production of germ cells. Previous studies have shown that ESC-derived PGC-like cells and germ cells have been spontaneously or directly differentiated by applying RA and/or BMP4 in culture, but a substantial number of these differentiated cells expressed specific markers for PGCs or germ cells only *in vitro* [3]–[4], [9]. In the present study, we applied an efficient three-step sequential method for inducing and propagating ESC-derived GSC-like cells and producing haploid male germ cells in *in vitro* systems. The results have confirmed that the addition of RA and BMP4 to cultures effectively induces the expression of GSC-specific markers in differentiated hESCs [30]–[32]. In addition, more enriched ESC-derived GSC-like cells were obtained using a germ cell culture system, and the efficiency of haploid male germ cell production was increased using a 3D-co-culture system as compared to a 3D culture system alone (Fig. 5) and a 2D culture system(<1%, data not shown). Propagated ESC-derived GSC-like cells maintained the potential for proliferation capacity, as confirmed through cell transplantation into the testes of host mice (Fig. S6). Moreover, *in vitro* differentiated GSC-like cells have shown similar gene expression profiles in SSCs obtained from human testes.

To improve low differentiation efficiency, we identified the critical factors involved in the differentiation of GSCs into germ cells [33]–[34] and the proliferation of *in vitro* induced GSC-like cells. Indeed, the addition of BMP and RA has shown that some growth factors might affect the induction of differentiation into GSC-like cells and the enrichment of GSC-like cells *in vitro*. Several groups have reported that mouse GSC clumps continuously proliferate under culture conditions with GDNF and bFGF, and these proliferating clump-forming cells maintain the properties of GSCs [20], [35]. In human systems, GSC-like cells proliferate when certain growth factors are added to the culture medium [23]. In the present study, we applied GC-proliferation medium supplemented with EGF, LIF, GDNF and bFGF to propagate and enrich ESC-derived GSC-like cells, and the population of GFR-α1-positive cells derived from cultured EBs was highly enriched compared with the control group (Fig. 3).

In our previous studies, we introduced an *in vitro* 3D-culture system using sodium alginate for differentiation into haploid male germ cells [21]–[22]. For the effective differentiation of GSC-like cells derived from human ESCs, we employed a modified 3D-co-culture system using testicular somatic cells (Fig. 4). After *in vitro* differentiation and quantification using markers for haploid male germ cells, we observed that a 3D-co-culture system was more effective than a simple 3D-culture system for the differentiation of GSC-like cells from human ESCs (Fig. 5). In particular, approximately 3% of the GSC-like cells could be differentiated into haploid male germ cells, exhibiting specific markers after only two weeks of short-term culture, although morphologically normal or functional spermatozoa were not observed in the cell cultures.

GFR-α1 is the receptor for GDNF, a growth factor that regulates the ratio of self-renewal and differentiation of GSCs. GDNF-knockout mice exhibited a depletion of GSCs, whereas mice over-expressing GDNF show an accumulation of GSCs. von Schonfeldt and colleagues reported that GFR-α1 is localized to spermatogonia in the adult mouse testis [29], suggesting that male GSCs could be highly purified using this marker. Although CD9 and THY-1 are generally accepted to be male GSC markers, these surface markers were also detected on undifferentiated stem cells. In the present study, GFR-α1 was used as a marker for sorting male GSCs, and the sorted GFR-α1-positive cells were cultured (Fig. 3). In contrast to previous studies showing a 16 to 32-fold expansion after culturing for 1 month [20], the expansion rate of our GSC-like cells was low (3–4-fold). The results suggest that these cells might be different from other GSCs obtained from the testes, although GSC-like cells derived from hESCs exhibited similarities in morphology and marker expression of human male GSCs. Therefore, we compared the gene expression profiles of ESC-derived GSC-like cells and male GSCs (spermatogonia) obtained from human testes [23] using microarray analysis. The gene sets over-expressed in GSC-like cells derived from hESCs showed a significant overlap with the reference gene lists used to characterize male GSC-specific genes in previous rodent studies [36]. The previously identified male GSC-specific genes, such as *ITGA6*, *FGFR3*, *EPB41L4A*, *DPPA5*, *DPPA3 (STELLA)*, *CHEK2*, *CHD7*, *CDH1*, *BCL6B*, *ABCG2*, *TERT*, *TACSTD1*, *SOX3*, *SOX2*, *SOHLH2*, *SEMA4D*, *RELN*, *PUNC*, *PIWIL2*, *NOTCH1*, *LIN28B*, and *LHX1*, showed similar expression levels in all human and rodent samples. However, some SSC-specific genes, such as *UPK1B*, *SOHLH1*, *SNAP91*, *TEX14*, *TEX101*, *NANOS2*, *MBP*, *DAZL*, *MAGEA4* and *STAR8*, were not expressed in either human sample (data not shown). The absence of those genes could reflect the fact that the human samples (GSC-like cells from hESCs and testicular spermatogonia) did not exhibit the same gene expression patterns as the rodent samples. Using microarray analysis, we identified six male GSC-specific genes showing differential expression in both human samples: the expression of *TSPAN8* and *FOXA2* in the testis and the expression of *SLC6AL*, *FOXA2*, *ANKRD6* and *ABCG2* in GSC-like cells from hESCs. The results revealed that cultured GSC-like cells derived from hESCs have high similarity with human spermatogonia (Fig. 6A).

In the validated gene analysis (Fig. 6B), *protein tyrosine phosphatases (PTPs)*, namely, *DUSP6*, *PTPN14*, *PTPRG*, *PTPRM*, and *PTPRD*, regulate adhesion and differentiation. *Dual specific phosphatase 6 (DUSP6* or *MKP*-3) encodes a negative feedback regulator of ERK signaling and regulates FGFR signaling during mouse development. *PTPRM* is associated with adherent junctions and dephosphorylates key signaling substrates, such as beta-catenin and cadherin adhesion molecules. *PTPRG* is transiently expressed during ESC-derived embryoid body differentiation and is required for male GSC lineage commitment [37]–[39]. *TENC1* encodes a member of the tensin family that is a focal adhesion molecule that binds to actin filaments (ENC1) and participates in signaling pathways [40]. *NID2* encodes a member of the nidogen family, and this protein is a cell-adhesion protein that binds collagen I and IV and laminin and might be involved in maintaining the structure of the basement membrane. These genes have been associated with the formation of male GSC clumps and showed similar expression levels in ESC-derived GSC-like cells. *STARD13* encodes a protein that contains an N-terminal sterile alpha motif (SAM) for protein-protein interactions followed by an ATP/GTP-binding motif, a GTPase-activating protein (GAP) domain, and a C-terminal STAR-related lipid transfer (START) domain, suggesting that this protein might be involved in the regulation of cell proliferation. In addition, *STARD13* interacts with EPB41L and TENC1 [40]–[41]. These genes have been associated with the proliferation of male GSC clumps and exhibited lower expression in GSC-like cells from hESCs than in male GSCs from testes, potentially reflecting the low expansion rate of GSC-like cells. *NR0B1* encodes an orphan member of the nuclear hormone receptor family that is expressed in tissues involved in the production of steroid hormones and the reproductive function of the testes. Indeed, NR0B1 null homozygous male mice have adrenal insufficiency and testicular disorganization, as well as failed spermatogenesis [42]–[43]. All of the above genes are associated with spermatogenesis, reflecting the fact that ESC-derived GSC-like cells are more similar to germ cells than to undifferentiated hESCs.

## Conclusions

We developed a sequential culture system to induce and propagate human GSC-like cells that strongly express the male GSC-specific maker GFR-α1 and differentiate into male germ cells. Although the GSC-like cells derived from hESCs were not identical to spermatogonia, this study provided insight into the induction and propagation of spermatogonia. In addition, a large number of GSC-like cells can be applied to 3D-co-culture for differentiation into male germ cells. Therefore, we suggest that a sequential differentiation system is a useful tool for the study of male germ cell development and for future clinical trials using patient specific stem cells after more improvement of male GSC induction and the completion of meiosis.

## Supporting Information

Figure S1 Schematic representation of the three-step method.
(JPG)

Figure S2 Characterization of undifferentiated human embryonic stem cells (CHA-hES15) Immunocytochemical staining for OCT4, NANOG, SSEA-3, SSEA-4 and TRA1-60, as human ESC-specific markers, and staining for VASA and GFR-α1, as spermatogonia specific markers. The bright field images show the typical morphology of hESCs. Scale bar is 100 µm.
(JPG)

Figure S3 Characterization of human spermatogonial cells. (A) Cellular localization of VASA and GFR-α1 as spermatogonia specific markers in fetal gonads and adult testes. VASA was abundantly expressed in fetal gonad cells. GFR-α1 was observed in the undifferentiated spermatogonial cells at the basement membrane within the seminiferous tubules. The figure on the right shows the magnification of VASA and GFR-α1-positive signals. Scale bars: 50 µm. (B) Immunocytochemical characterization of cultured spermatogonial cells using human adult testes. Integrin β1, α6 and GFR-α1 were used as spermatogonia markers. The bright field images show the typical morphology of cultured spermatogonial cells. OA testis: seminiferous tubules of OA (normal spermatogenesis) patients. Scale bars: 50 µm. (C) RT-PCR-based characterization of hESCs, GSC-like cells and spermatogonial cells. Note: Mock 1^st^ ab: not treated with primary antibodies, MEF: mouse embryonic fibroblast.
(JPG)

Figure S4 Expression profiles of undifferentiated hESCs, hESC-derived GSC-like cells and testis-derived spermatogonial cells. The expression profiles of the 9534/42404 genes that were differentially expressed in the three types of cells were hierarchically clustered and are presented as a heat-map. The expression level of each transcript is indicated in the color code bar; red indicates high expression, and green indicates low expression. Note: hESCs, undifferentiated human embryonic stem cells; GSC-like cells (from hESCs), *in vitro* cultured human ESC-derived GSC-like cells at 2^nd^ passage; spermatogonia (Testis), *in vitro* cultured testis-derived spermatogonial cells at 2^nd^ passage.
(JPG)

Figure S5 Differentially expressed gene profiles of undifferentiated hESCs, hESC-derived GSC-like cells and testis-derived spermatogonial cells. (A) The expression profiles of the 9534 (count of differentially expressed genes (DEG) and their hierarchical clustering) genes that were expressed in the 3 types of cells. (B) Functional classification using gene ontology information. Note: hESCs, undifferentiated human embryonic stem cells; GSC-like cells (from hESCs), *in vitro* cultured human ESC-derived GSC-like cells at 2^nd^ passage; spermatogonia (Testis), *in vitro* cultured testis-derived spermatogonial cells at 2^nd^ passage.
(JPG)

Figure S6 In vivo propagation of GSC-like cells in recipient testis (A) Testes after transplantation with GSC-like cells using injection pipettes. GSC-like cells were suspended in DPBS containing trypan blue. Seminiferous tubules containing the blue cell suspension were observed. (B) GFP signaling in GSC-like cells from recipient testes. Scale bars: 50 µm.
(JPG)

Figure S7 Different type of FISH results. Detection of X chromosome and 9 chromosome in the differentiated GSC-like cells; a and a′: diploid (2n), b and b′: tetraploid (4n), c and c′: haploid (n,X) d and d′: haploid type (n,Y); Upper panel indicated CHA-hES15 cell lines. Lower panel indicated H1 cell lines.
(JPG)
