# Supplementary figures and images for: Three-Step Method for Proliferation and Differentiation of Human Embryonic Stem Cell (hESC)-Derived Male Germ Cells
Source: PLoS One. 2014 Apr 1;9(4):e90454. doi: 10.1371/journal.pone.0090454 (PMC3972183; doi:10.1371/journal.pone.0090454)

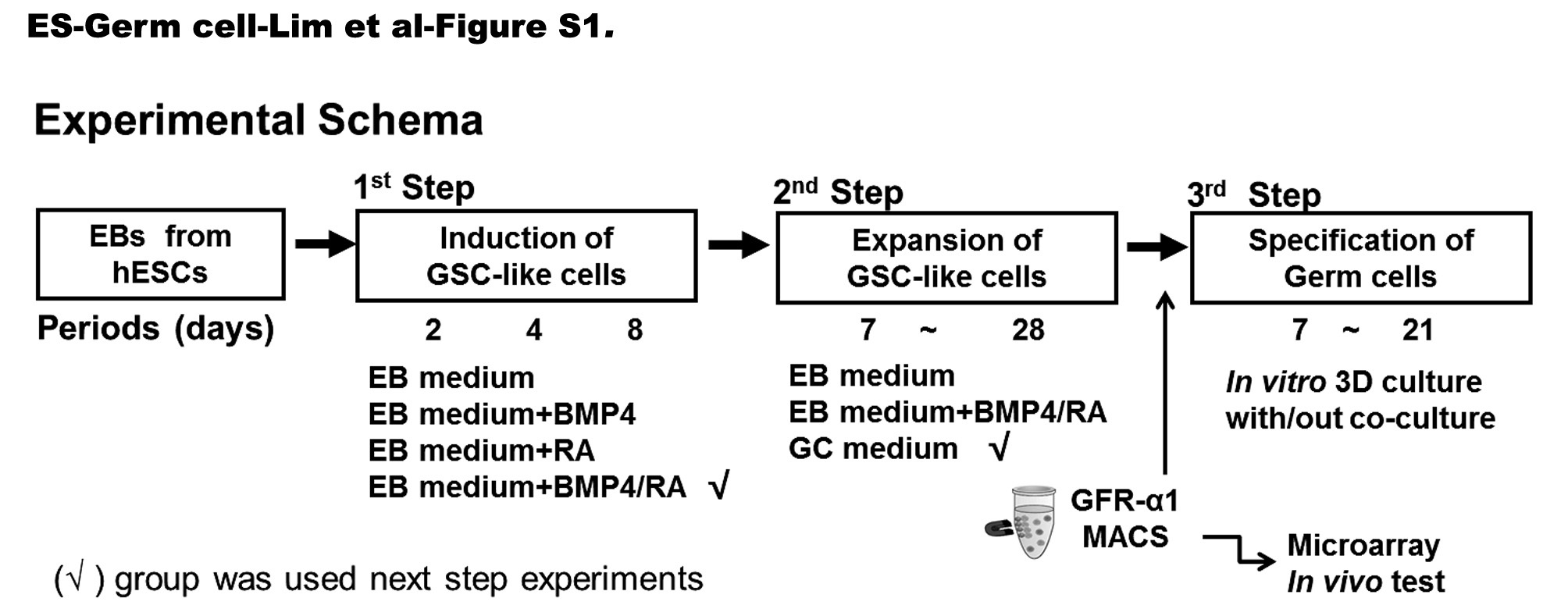

Supplement: Figure S1 — Schematic representation of the three-step method. (JPG) [file pone.0090454.s001.jpg]

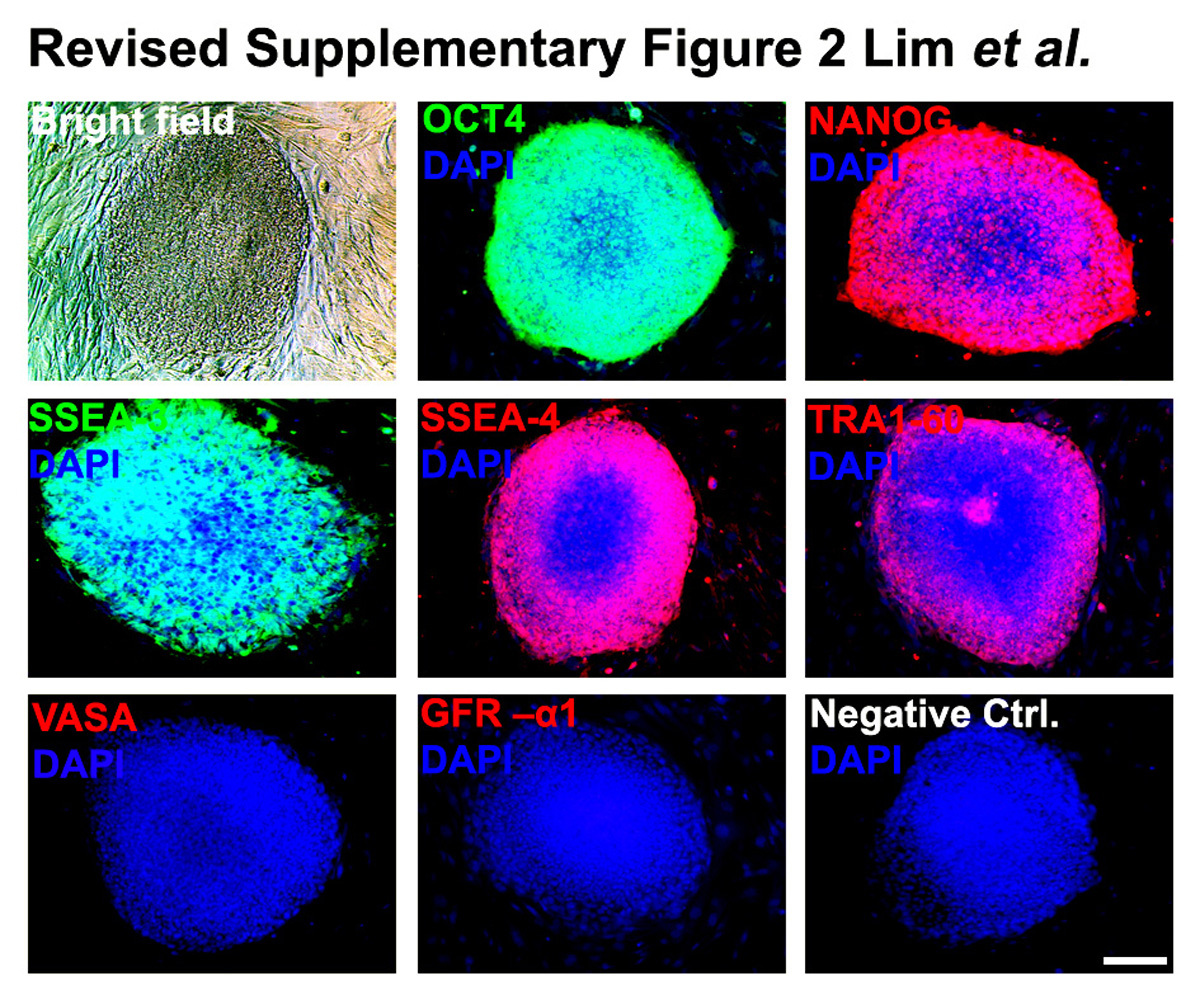

Supplement: Figure S2 — Characterization of undifferentiated human embryonic stem cells (CHA-hES15) Immunocytochemical staining for OCT4, NANOG, SSEA-3, SSEA-4 and TRA1-60, as human ESC-specific markers, and staining for VASA and GFR-α1, as spermatogonia specific markers. The bright field images show the typical morphology of hESCs. Scale bar is 100 µm. (JPG) [file pone.0090454.s002.jpg]

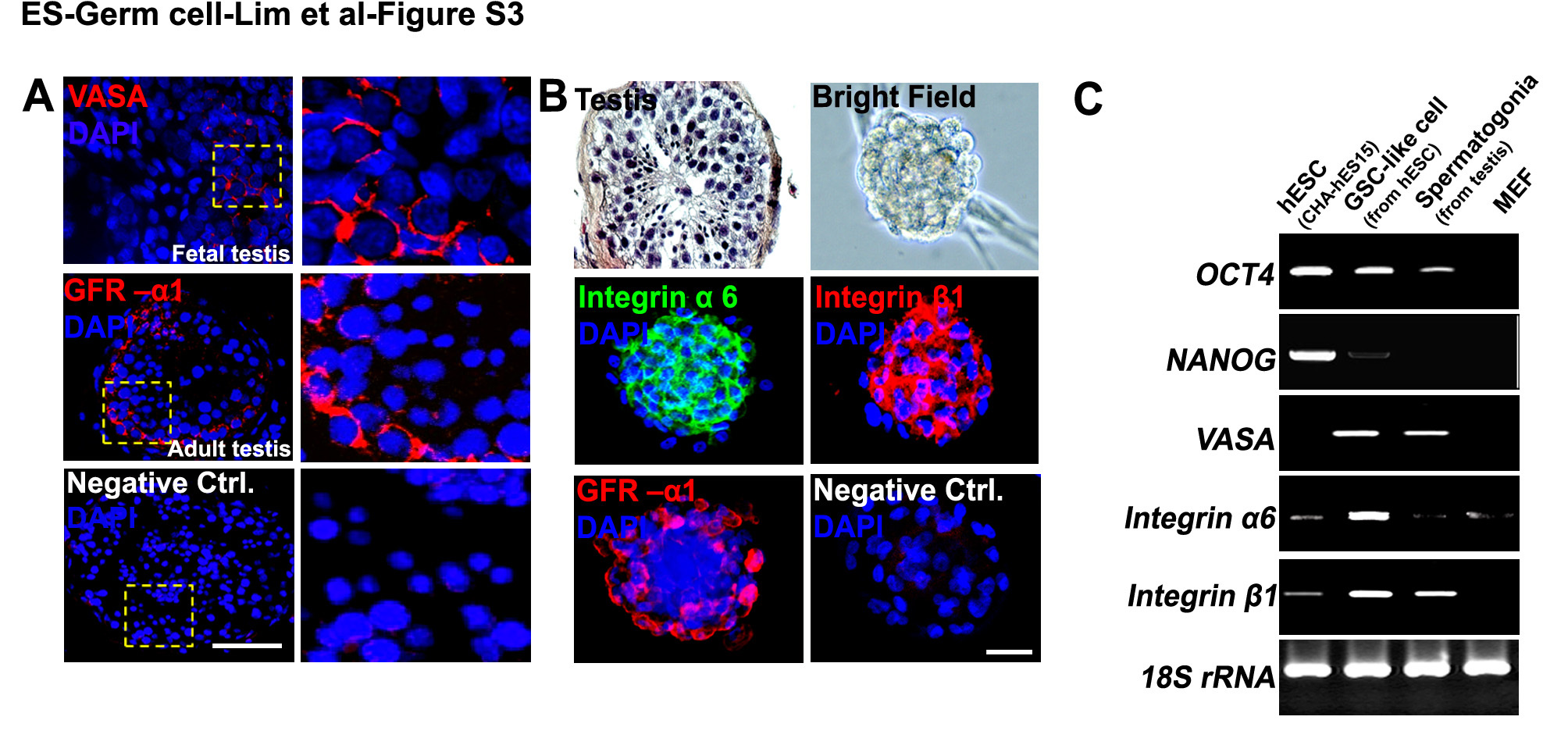

Supplement: Figure S3 — Characterization of human spermatogonial cells. (A) Cellular localization of VASA and GFR-α1 as spermatogonia specific markers in fetal gonads and adult testes. VASA was abundantly expressed in fetal gonad cells. GFR-α1 was observed in the undifferentiated spermatogonial cells at the basement membrane within the seminiferous tubules. The figure on the right shows the magnification of VASA and GFR-α1-positive signals. Scale bars: 50 µm. (B) Immunocytochemical characterization of cultured spermatogonial cells using human adult testes. Integrin β1, α6 and GFR-α1 were used as spermatogonia markers. The bright field images show the typical morphology of cultured spermatogonial cells. OA testis: seminiferous tubules of OA (normal spermatogenesis) patients. Scale bars: 50 µm. (C) RT-PCR-based characterization of hESCs, GSC-like cells and spermatogonial cells. Note: Mock 1st ab: not treated with primary antibodies, MEF: mouse embryonic fibroblast. (JPG) [file pone.0090454.s003.jpg]

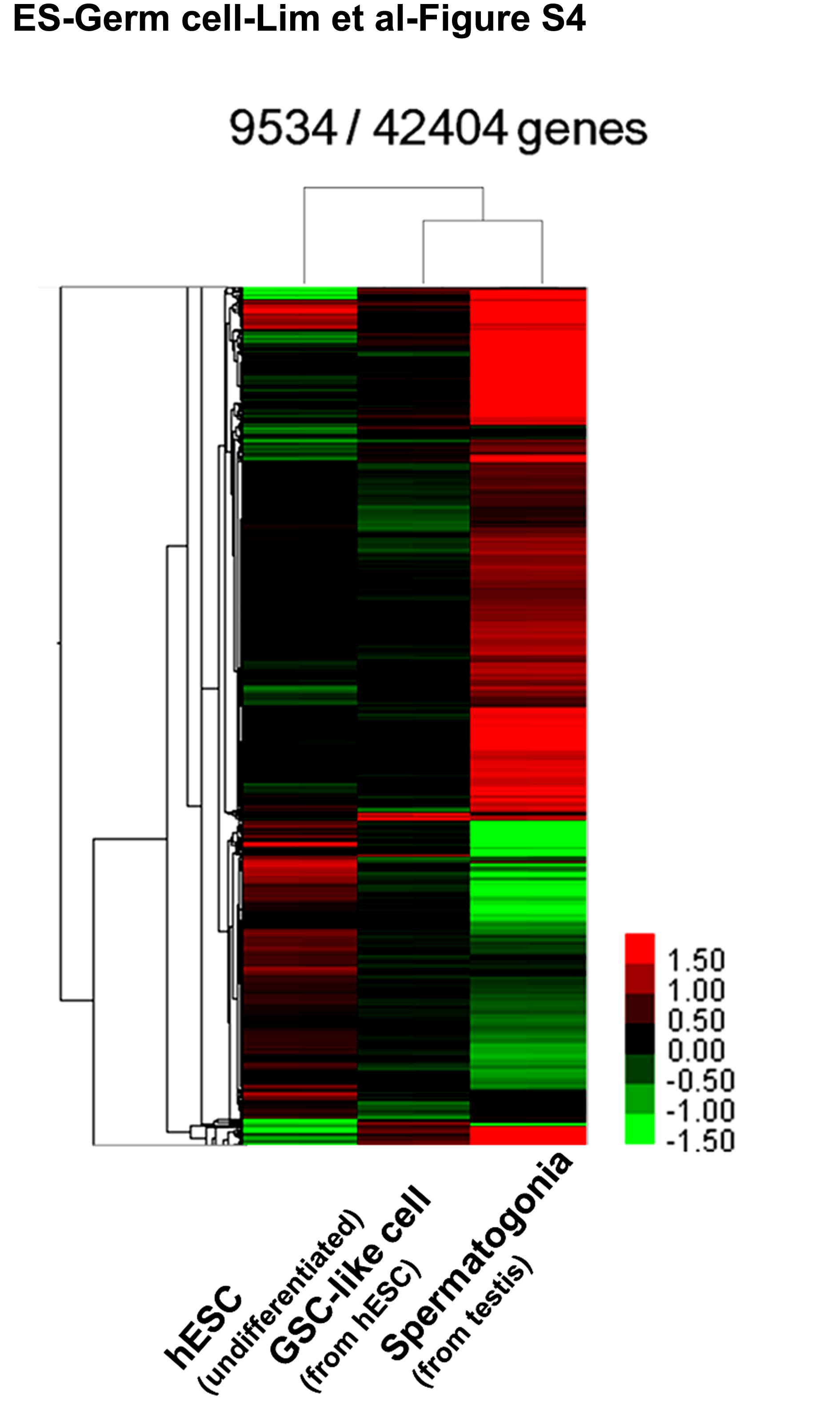

Supplement: Figure S4 — Expression profiles of undifferentiated hESCs, hESC-derived GSC-like cells and testis-derived spermatogonial cells. The expression profiles of the 9534/42404 genes that were differentially expressed in the three types of cells were hierarchically clustered and are presented as a heat-map. The expression level of each transcript is indicated in the color code bar; red indicates high expression, and green indicates low expression. Note: hESCs, undifferentiated human embryonic stem cells; GSC-like cells (from hESCs), in vitro cultured human ESC-derived GSC-like cells at 2nd passage; spermatogonia (Testis), in vitro cultured testis-derived spermatogonial cells at 2nd passage. (JPG) [file pone.0090454.s004.jpg]

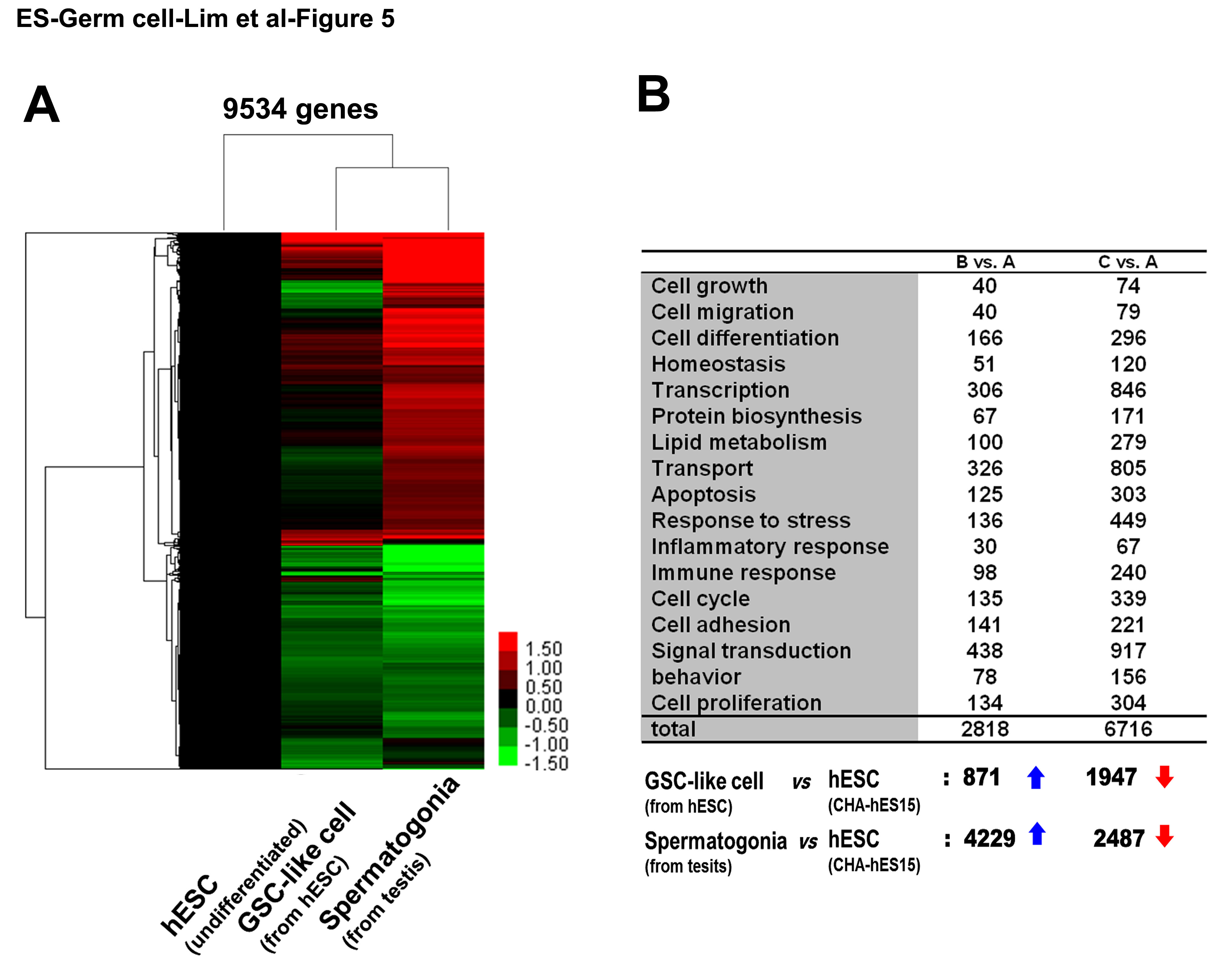

Supplement: Figure S5 — Differentially expressed gene profiles of undifferentiated hESCs, hESC-derived GSC-like cells and testis-derived spermatogonial cells. (A) The expression profiles of the 9534 (count of differentially expressed genes (DEG) and their hierarchical clustering) genes that were expressed in the 3 types of cells. (B) Functional classification using gene ontology information. Note: hESCs, undifferentiated human embryonic stem cells; GSC-like cells (from hESCs), in vitro cultured human ESC-derived GSC-like cells at 2nd passage; spermatogonia (Testis), in vitro cultured testis-derived spermatogonial cells at 2nd passage. (JPG) [file pone.0090454.s005.jpg]

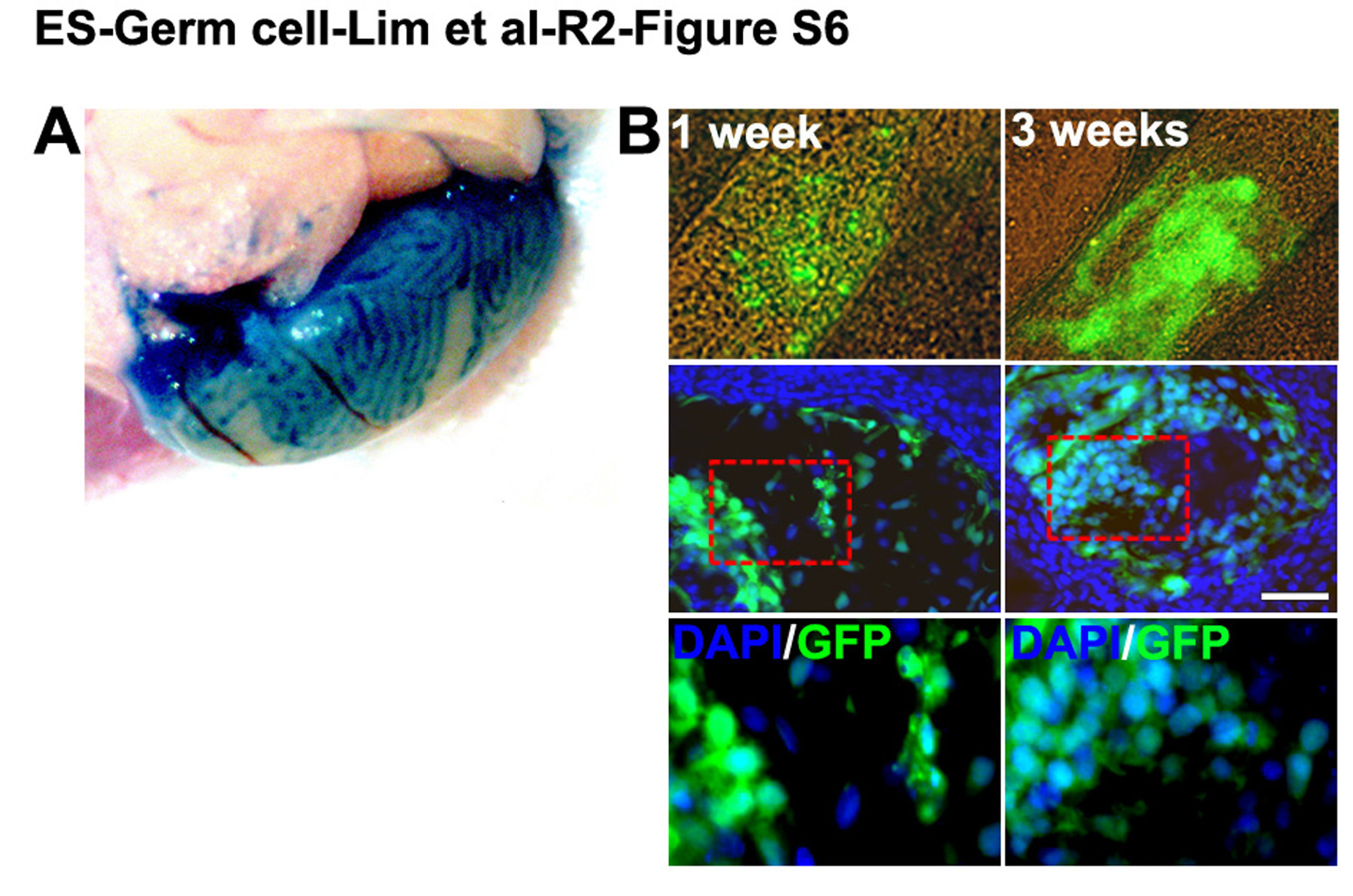

Supplement: Figure S6 — In vivo propagation of GSC-like cells in recipient testis (A) Testes after transplantation with GSC-like cells using injection pipettes. GSC-like cells were suspended in DPBS containing trypan blue. Seminiferous tubules containing the blue cell suspension were observed. (B) GFP signaling in GSC-like cells from recipient testes. Scale bars: 50 µm. (JPG) [file pone.0090454.s006.jpg]

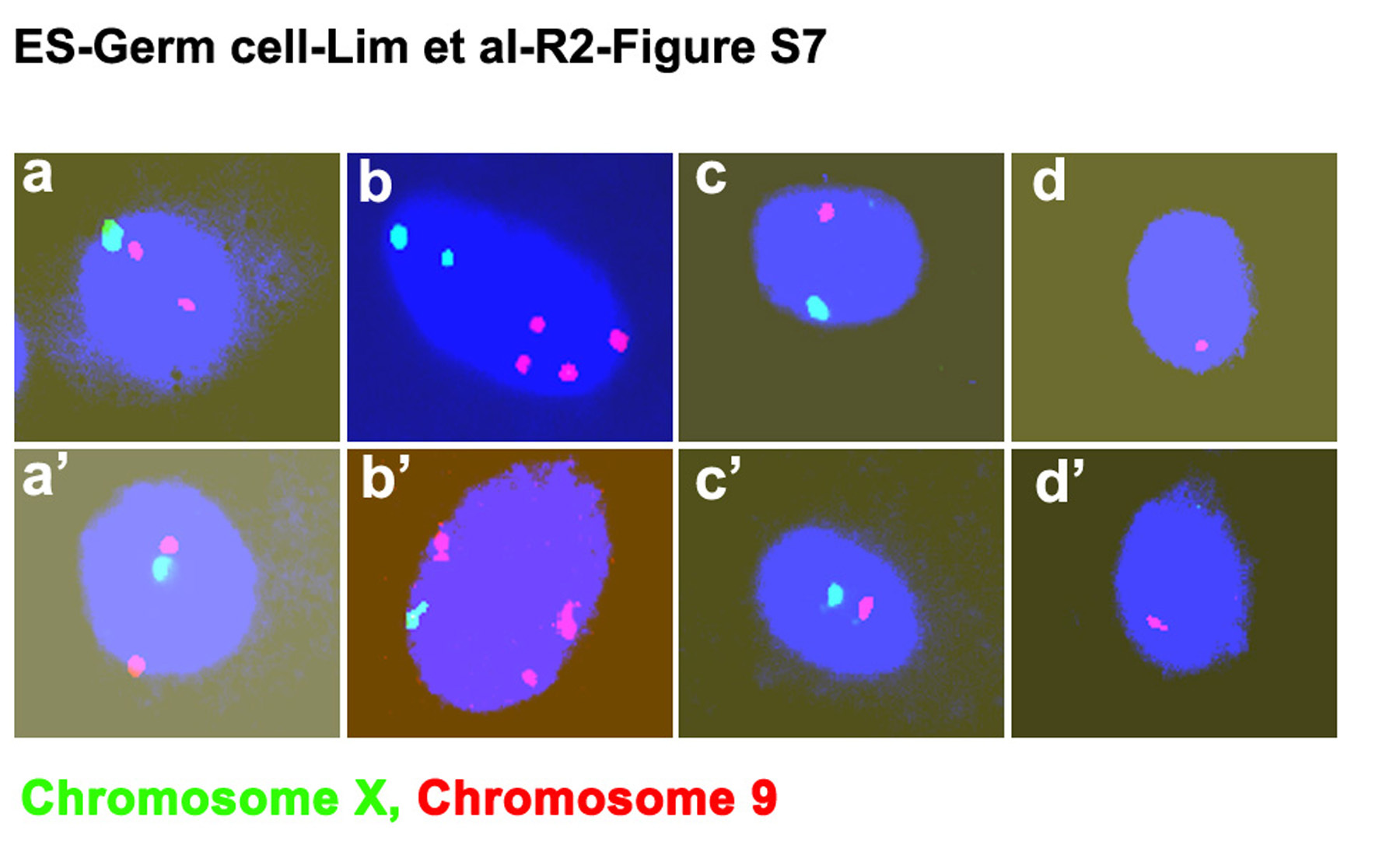

Supplement: Figure S7 — Different type of FISH results. Detection of X chromosome and 9 chromosome in the differentiated GSC-like cells; a and a′: diploid (2n), b and b′: tetraploid (4n), c and c′: haploid (n,X) d and d′: haploid type (n,Y); Upper panel indicated CHA-hES15 cell lines. Lower panel indicated H1 cell lines. (JPG) [file pone.0090454.s007.jpg]
